# Development of a single resistance to damage metric for mosquito nets related to physical integrity in the field

**DOI:** 10.1186/s12936-020-03566-1

**Published:** 2021-01-19

**Authors:** Amy Wheldrake, Estelle Guillemois, Vera Chetty, Albert Kilian, Stephen J. Russell

**Affiliations:** 1 Nonwovens Innovation & Research Institute Ltd, 169 Meanwood Road, Leeds, LS7 1SR West Yorkshire UK; 2 Tropical Health LLP, Montagut, Spain

**Keywords:** Long-lasting insecticidal mosquito nets, Resistance to damage, Physical integrity, Durability

## Abstract

**Background:**

In common with the majority of personal protective equipment and healthcare products, the ability for long-lasting insecticidal nets (LLINs) to remain in good physical condition during use is a key factor governing fitness for purpose and serviceability. The inherent ability of a product to resist physical deterioration should be known in advance of it being used to ensure it has maximum value to both the end-user and procurer. The objective of this study was to develop a single performance metric of resistance to damage (RD) that can be applied to any LLIN product prior to distribution.

**Methods:**

Algorithms to calculate RD values were developed based on consideration of both human factors and laboratory testing data. Quantitative reference forces applied to LLINs by users during normal use were determined so that aspirational performance levels could be established. The ability of LLINs to resist mechanical damage was assessed based on a new suite of textile tests, reflecting actual mechanisms of physical deterioration during normal household use. These tests quantified the snag strength, bursting strength, abrasion resistance and resistance to hole enlargement. Sixteen different unused LLINs were included in the analysis. The calculated RD values for all LLINs and the corresponding physical integrity data for the same nets retrieved from the field (up to 3 years of use) were then compared.

**Results:**

On a RD scale of 0 (lowest resistance) – 100 (highest resistance), only six of the sixteen LLINs achieved an RD value above 50. No current LLIN achieved the aspirational level of resistance to damage (RD = 100), suggesting that product innovation is urgently required to increase the RD of LLINs. LLINs with higher RD values were associated with lower hole damage (PHI) in the field when adjusted for normal use conditions.

**Conclusions:**

The RD value of any LLIN product can be determined prior to distribution based on the developed algorithms and laboratory textile testing data. Generally, LLINs need to achieve higher RD values to improve their ability to resist hole formation during normal use. Innovation in LLIN product design focused on the textile material should be actively encouraged and is urgently needed to close the performance gap.

## Background

In terms of reliability engineering, long-lasting insecticidal nets (LLINs) should be capable of functioning under field conditions for 3 or more years, and resist failure during normal use by remaining in good physical condition [1]. However, it is well known that the actual service life of LLINs can fall markedly short of 3 years, depending on prevailing circumstances. Whilst there have been numerous long-term studies monitoring the physical integrity and durability of LLINs using various methodologies [2–8], much less attention has been paid to the design of the LLIN product itself, and the inherent ability of products to resist damage. Therefore, at present, there is no single metric that reliably defines the inherent resistance to damage of a new LLIN before it is used.

Based on detailed laboratory analyses of used LLINs [9], distinct mechanisms of structural damage take place in LLINs that are common across different geographic regions and brands. Mechanical damage is the primary contributor to LLIN deterioration in normal use, both in terms of hole frequency and area. Of these mechanical damage mechanisms, snagging is responsible for the initiation of the largest proportion of holes. Although such holes may be initially small in dimensions, they can potentially enlarge to form significantly larger holes over time. Collectively, tearing, abrasion and seam failure are also responsible for a large proportion of the hole area, and their underlying mechanisms of damage would be practically difficult to avoid during normal use of a LLIN product. Tears usually form as a result of the net first being snagged on a solid object, such as wooden mattress material, and then when force is applied to pull it free, a tear is created. Abrasion occurs when two surfaces are rubbed against each other e.g. during washing or when the LLINs are tucked between the mattress and bedframe. Furthermore, forms of mechanical damage are recurrent across different geographical settings and are found in all knitted LLINs regardless of whether they are made of polyester (PET) or polyethylene (PE) [10, 11].

Studies have subsequently focused on developing textile testing methods for LLINs that relate to the actual damage sustained in the field [12]. Test method selection was based on identifying suitable test methods that accurately reflect the physical damage observed in LLINs analysed after use in the field [13]. Overall, four textile test methods based on existing or slightly modified ISO standards, have been proposed for LLINs to reflect actual modes of damage observed in the field. One of the proposed methods, i.e. the bursting test, is already used in the specification of LLIN products [14]. The other three methods are snag strength, hole enlargement and abrasion resistance [12]. This suite of four laboratory textile test methods currently yields four separate quantitative values to represent the inherent resistance to damage of LLINs.

The aim of the present study was to develop a single quantitative metric to define the resistance to damage (RD) of a LLIN based on the same laboratory test data, but which also considered the question ‘how strong is strong enough?’. This formed the basis to develop a new algorithm for calculating RD for new LLINs that should assist with future innovation and the development of better performing products.

## Methods

### Establishing real‐life forces

Over the course of normal use, LLINs accumulate physical damage that produce holes. For example, LLINs are frequently snagged on rigid objects, such as bits of wood, and when pulled by the user to free the snag, a force is generated. Little is known about the magnitude of the forces that are generated in such practical circumstances, and the levels required to induce filament breakages or create a tear. Simply stated, if the force measured in the lab under simulated conditions is greater than what can be normally generated by a human being in the field, then the LLIN can be expected to resist the accumulation of damage.

Such human factors and associated real-life forces and mechanisms of damage are routinely considered in the development of other protective and healthcare products to ensure products are fit for purpose. Table 1 indicates the maximum isometric forces that are capable of being safely exerted by a person [15]. According to this standard, a person in a seated position exerting a one-handed arm only pulling movement in a downwards direction, produces a maximum isometric force of 75 N. These values relate to isometric forces that can be steadily exerted while the limbs are in a stationary position [16]. Under field conditions, forces will also occur as a result of explosive strength, i.e. a dynamic burst of movement, and in these instances the forces capable of being exerted by a user are likely to be higher [15].

**Table 1:** Maximal isometric force measurements from EN 1005-3:2002 Safety of machinery [15]

Activity		Professional use F_a_ in N	Domestic use F_a_ in N
	Hand work (one hand): power grip	250	184
	Arm work (sitting posture, one arm):		
	upwards	50	31
	downwards	75	44
	outwards	55	31
	inwards	75	49
	pushing:		
	with trunk support	275	186
	without trunk support	62	30
	pulling:		
	with trunk support	225	169
	without trunk support	55	28

The maximum explosive forces that can be generated by humans have been studied by other researchers with the aim of informing designers about the safe design of products [17]. Pulling strength was one of the aspects studied and the values obtained are shown in Table 2. Note that the forces generated are much higher than those obtained for a person in a stationary position.

**Table 2:** Minimum and maximum mean force for overhand and underhand explosive

	Male aged 21–60 Min-Max mean force (N)	Female aged 21–60 Min-Max mean force (N)
Underhand pulling	145–185	85–123
Overhand pulling	99–308	78–260

**Fig. 1:**
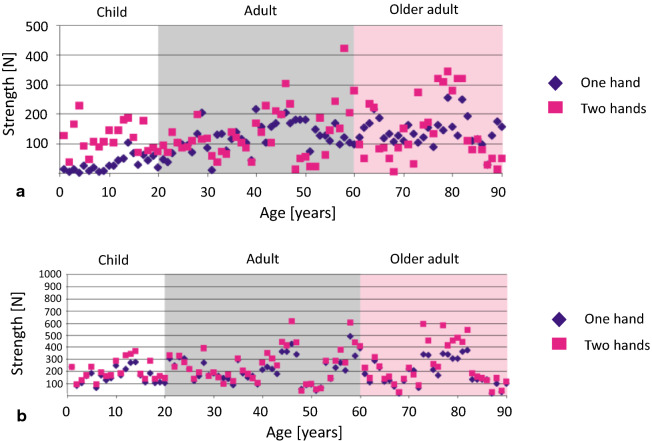
Maximum pulling strength **a** using an underhand grip and **b** on a round handle using an overhand grip [17]

The mean weight of adult males taking part in the experiment was 80 kg, while that for the women was 68 kg. This is higher than the average weight of adults in malaria endemic countries (Table 3) [18]. Strength has long been established as being influenced, by body weight as well as other factors [19]. Therefore, this should be reflected when setting an aspirational performance target for the strength or resistance to damage of a LLIN based on published real life forces. Forces recorded in Newtons (N) are straightforward to relate to the snag strength test in the laboratory, which measures the force (N) required to break a yarn in the LLIN. Furthermore, data on the forces generated during underhand or overhand pulling is available (Fig. 1) [17].

**Table 3:** Mean body weight of adult humans in different malaria-endemic countries

Mean weight of adult human	
Country	kg
India	52.9
Ethiopia	53.1
Congo	53.5
Cambodia	55.7
Mozambique	56
Kenya	56.3
Uganda	57
Benin	60.3
Nigeria	60.7
All of World	62

### LLIN samples for testing

Sixteen different new and unused LLIN products supplied by 10 different brands representing the majority of WHOPES (World Health Organization Pesticide Evaluation Scheme) recommended insecticide treated nets (ITNs) in 2013 were obtained from suppliers to allow textile testing data to be generated for snag strength, bursting strength, abrasion resistance and hole enlargement. Details of the sample preparation, textile testing procedures and number of replicates have been previously reported [12]. The datasets for each LLIN were then used to calculate a RD value based on the developed algorithm.

#### Algorithm development

Approaches for predicting the service life of products, product performance during use, and warranty costs [20, 21] are all well established. Methods to predict service life of products rely on mathematical models based on prior knowledge of the appropriate distribution that should be used, as well as data on how users and the environment interact with products in the field [22]. Given the complexities involved in such an approach with LLINs, a criterion-referenced assessment of the resistance to damage of LLINs was applied based upon the quantitative values obtained for each durability test parameter and human factors, specifically based on the magnitude of forces that is likely to be applied to a LLIN by its user. Two different methods were developed for determining the RD values for LLINs.

##### Method 1: Proximity to aspirational values

In this method, laboratory testing data for snag strength, bursting strength, abrasion and hole enlargement, i.e. actual values (*λ*), were compared with aspirational values (*ɳ*) for each parameter to determine numerical differences in performance. The mean value for each parameter was then divided by four so that each contributed equally to the overall RD value, expressed as a percentage (Eq. 1):

1 $$\text{R}\text{D}=\left( \frac{{\lambda }_{B}}{{\eta}_{B}} \times \frac{100}{4}\right) + \left( \frac{{\lambda }_{S}}{{\eta}_{S}} \times \frac{100}{4}\right)+ \left( \frac{{\lambda }_{A}}{{\eta}_{A}} \times \frac{100}{4}\right)+\frac{{\sigma }_{H}}{4}$$

where: RD = Resistance to damage; ${\uplambda }_{B}$ = Actual bursting strength (kPa);  ${\eta}_{B}$ = Aspirational bursting strength (kPa); ${\lambda }_{S}$ = Actual snag strength (N); ${\eta}_{S}$ = Aspirational snag strength (N); ${\lambda }_{A}$ = Actual abrasion resistance strength (number of rubs); ${\eta}_{A}$ = Aspirational abrasion resistance (number of rubs); σ_H_ = Hole enlargement resistance score.

The hole enlargement value for Eq. 1 was defined by a score, which was based on the final hole size obtained in the hole enlargement laboratory test and the presence (or not) of hole enlargement due to laddering, unravelling or tearing, as defined in Table 4.

**Table 4:** Hole enlargement resistance scores reflecting the hole size and type of hole enlargement for Method 1

Damage type	End hole size		
	< 5 mm	6–20 mm	≥ 21 mm
None	100	80	40
Laddering	80	64	32
Unravelling	50	40	20
Tearing combined with laddering or unravelling	40	32	16

### Algorithm development-method 2: proximity to aspirational value by KPI

Method 2 is based on a Key Performance Indicator system (KPI), which uses the laboratory data to establish scores for each of the four individual test parameters. The basic approach is used across a range of sectors including healthcare, performance monitoring in the business sector, education and customer services [23, 24]. As the name suggests, the method measures performance against pre-specified criteria based on aspirational values defined for each test parameter. The methodology allows for qualitative data to be included and provides a solution for dealing with multi-objective outcomes. It does this by use of a scoring matrix. The score matrix structure is illustrated in Table 5. For each test parameter, the matrix defines test value ranges up to the aspirational value in equal proportions. The overall score for bursting strength, snag strength, abrasion resistance and hole enlargement contribute equally to the overall RD value.

**Table 5:** Score matrix for calculation of RD values including hole enlargement using Method 2

Bursting Strength (kPa)	Lowest test value range from lab	Intermediate test value ranges																			Highest test value range from lab
Bursting Strength Score	1	2.5	5			7.5		10	12.5			15		17.5		20			22.5		25
Snag Strength (N)	Lowest test value range from lab	Intermediate test value ranges																			Highest test value range from lab
Snag Strength Score	1	2.5	5			7.5		10	12.5			15		17.5		20			22.5		25
Abrasion Resistance (rubs)	Lowest test value range from lab	Intermediate test value ranges																		Highest test value range from lab	
Abrasion Resistance Score	1	3		6			9			13			16		19			22		25	
Hole Enlargement (mm)	≥ 21 mm						20 − 6 mm								≤ 5 mm						
Hole Enlargement Resistance Score	10						20								25						
Secondary Damage	Laddering				Unravelling												Tearing combined with Laddering or Unravelling				
Secondary Damage Penalty	10				20												30				
Penalty Score	Secondary damage penalty x Hole enlargement resistance score/100																				

For the hole enlargement resistance score, a weighting is applied, as indicated in Table 4, which is based on the hole enlargement behaviour. These weightings are based on the analysis of field nets in the study by Wheldrake et al. [13], where laddering, unravelling and tearing were responsible for large holes, and as such their presence results in large weightings. In method 2, the overall RD value is, therefore, calculated as follows (Eq. 2).

2 $$RD = {\sigma }_{B}+ {\sigma }_{S}+ {\sigma }_{A} + ({\sigma }_{H} - {\rho }_{H})$$

where ${\sigma }_{B}$ is the score for bursting strength, ${\sigma }_{S}$ is the score for snag strength, ${\sigma }_{A}$ is the score for abrasion resistance, ${\sigma }_{H}$ is the score for hole enlargement resistance and ${\rho }_{H}$ is the penalty score.

### Correlation

The calculated RD values for all LLINs were determined based on the laboratory test data obtained from the suite of four textile tests [12] and the two methodological approaches (Methods 1 and 2) using equations 1 and 2. To explore any correlation between the RD data obtained in the lab and the extent of hole formation in the field, RD values for different LLINs were compared with previously reported [13] proportionate hole index (PHI) data after 1–3 years which uses the World Health Organization (WHO)-recommended metric for physical integrity in the field [25]. The proportionate hole index (PHI) data corresponds exclusively to holes that through forensic assessment were determined to have been the result of mechanical damage from reasonable household use, i.e. snagging, tearing and abrasion, and excluding cut, seam failure, rodent and thermal damage. The PHI for each LLIN was calculated following WHO guidelines as summarized in Table 6.

**Table 6:** WHO hole size guidelines and hole index used to assess physical integrity of LLINs

WHO 2013 guidelines	Size banding	Hole diameter	Hole radius		Area of hole	Hole Index^a^
	cm	d; cm	r = d/2; cm	r^2^; cm^2^	cm^2^	
Size 1 Smaller than a thumb	0.5–2	1.25	0.625	0.3906	1.23	1
Size 2 Larger than a thumb but smaller than a fist	2.5–10	6	3	9	28.28	23
Size 3 Larger than a fist but smaller than a head	11–25	17.5	8.75	76.5625	240.56	196
Size 4 Larger than a head	≥ 26	30^b^	15	225	706.95	576

A -area of the hole pr^2^; p = 3.142; ^a^ Area divided by 1.23; ^b^ Assumer diameter

Thus, if the weighting of the hole sizes 1, 2, 3 and 4 is α, β, γ and δ respectively, the hole index (H_i_) is calculated as in Eq. 3:

3 $${\text{H}}_{i} = \left({\upalpha }\times {\text{N}}_{1}\right)+\left({\upbeta } \times {\text{N}}_{2}\right)+\left({\upgamma } \times {\text{N}}_{3}\right)+\left({\updelta } \times {\text{N}}_{4}\right)$$

where N_1_ is the number of size 1 holes, N_2_ is the number of size 2 holes, N_3_ is the number of size 3 holes and N_4_ is the number of size 4 holes.

## Results

### Specification of aspirational values

#### Snag strength

The laboratory snag test is performed over a small surface area of the fabric ~ 1mm^2^ [12], which is consistent with the relatively small holes that this mechanism most frequently produces in real field nets [13]. The maximum explosive force a male of 21–60 years weighing an average of 80 kg can exert in an overhand pulling motion is reported to be 308 N [17] and therefore it is unlikely that a LLIN with snag strength of > 300 N would be readily damaged by snagging during normal use. Based on the results in Fig. 1, if a maximum upper limit, or effective reference value, was therefore placed at 200 N it would be greater than the maximum most of the expected users in malaria-endemic regions could exert on the LLIN in an underhand grip (Fig. 1a). In the case of overhand pulling (Fig. 1b), a proportion of the measured forces occur above 200 N, but it should be noted that this data set is based on a heavier adult population. Additionally, to verify the ease with which yarns in LLINs could be broken by human adults, manual snag tests were performed with a mixture of males and females aged between 27 and 42 years and weighing between 58 and 90 kg. LLINs with snag strength greater than 60 N required substantial effort by the participants to break the filaments. Thus, 200 N is considered a reasonable aspirational snag strength value.

#### Bursting strength

In Table 2, the mean maximum overhand pulling force that can be generated by an adult male aged 21–60 years is 308 N. Considering the 7.3 cm^2^ burst area, this is equivalent to 421 kPa. Therefore, in theory, a LLIN with a bursting strength > 421 kPa caught on a 7.3 cm^2^ surface and pulled with an underhand grip in an explosive manner, by a 21–60 years old adult male weighing ≤ 80 kg, should not easily break. This is based on assumptions such as the pressure being equally distributed across the surface area. In reality, LLINs with bursting strengths > 421 kPa have been found to contain tears [13]. This is likely to be due to nets being caught on smaller radius objects, which reduce the surface area over which the force acts. For example, if the same 308 N force was exerted over half the surface area of 3.65 cm^2^, the equivalent pressure would be 843 kPa. If the surface area was reduced further to represent a corner of a bed frame, e.g. 1 cm^2^ surface area, the equivalent pressure would increase to > 3000 kPa.

If the upper limit for force based on reasonable use is ca. 200 N [26] and this is applied to bursting strength using a 7.3 cm^2^ burst area, the corresponding pressure at break is 274 kPa. This is similar to the current WHO threshold value of 250 kPa [27]. However, the possibility of the surface area in the field being substantially smaller than 7.3 cm^2^ also needs to be taken into account, and, therefore, a safety factor needs to be built in. It is not possible to predict the possible surface areas that the net will interact with, which may lead to a failure, as these will be greatly variable in size, shape and surface texture. Generally, forces greater than 500 N would be at the upper limit of what is likely to be generated, which over a 7.3 cm^2^ area is equivalent to 685 kPa. Therefore, the aspirational level for the bursting strength of the nets was estimated at ≥ 700 kPa. Note that current burst testing equipment used by most textile testing laboratories record values up to a maximum of 1000 kPa.

### Abrasion resistance

Other industries that use an accelerated abrasion test with fine sandpaper as the abradant set a pass rate at 1000 rubs [28]. However, this is usually based on testing heavier-weight woven fabrics that are subjected to intensive flat abrasion during use. Therefore, based on the proportional difference in weight and in the mode of use of LLINs, an aspirational reference value of 400 rubs without failure was estimated.

### Hole enlargement

Current WHO guidelines recommend that hole sizes of 5 mm and greater are recorded [29]. This size is considered to be of practical relevance, given that the principal role of a LLIN is to act as a physical barrier to the penetration of mosquitoes. Therefore, it is consistent with current WHO guidelines to suggest an aspirational hole enlargement size of 5 mm following a yarn breakage, which is equivalent to a score of 100 for hole enlargement.

In the Proportionate Hole Index (PHI) used to determine the condition of field nets, size 1 holes are those between 5 and 20 mm. Herein, a penalty weighting was therefore introduced because of the need to account for LLINs that are susceptible to hole enlargement once yarns in the fabric are broken as a consequence of their knitting pattern. The hole enlargement resistance score due to secondary tears, laddering and unravelling are also accounted for in the RD calculation. The most serious secondary damage in terms of the size of holes formed is associated with combination of laddering and tearing or unravelling and tearing and this is reflected in the suggested weightings.

A summary of the aspirational values for each parameter comprising the RD value and used to calculate RD by method 1 and 2 (Eqs. 1 and 2) is given in Tables 7and 8.

**Table 7:** Definition of effective reference values for snag strength, bursting strength abrasion resistance and hole enlargement

	Textile Testing Parameter			
	Mean snag strength (N)	Mean bursting strength (kPa)	Abrasion resistance (number of rubs)	Hole enlargement resistance score
Aspirational value	≥ 200	≥ 700	400 (Allows 5 out of 15 to fail)	100 No Laddering No Unravelling No Tearing

**Table 8:** Score matrix and hole enlargement behaviour penalty for calculation of RD values using Method 2

Bursting Strength (kPa)	< 250	250–299		300–349			350–399		400–449	450–499		500–549		550–599		600–649			650 − 600	≥ 700
Bursting Strength Score	1	2.5		5			7.5		10	12.5		15		17.5		20			22.5	25
Snag Strength (N)	< 20	20–39		40–59			60–79		80–99	100–119		120–139		140–159		160–179			180–199	≥ 200
Snag Strength Score	1	2.5		5			7.5		10	12.5		15		17.5		20			22.5	25
Abrasion Resistance (rubs)	< 25		25–50		75–100			125–150		175–200			250		300			350		≥ 400
Abrasion Resistance Score	1		3		6			9		13			16		19			22		25
Hole Enlargement (mm)	≥ 21 mm							20 − 6 mm							≤ 5 mm					
Hole Enlargement Resistance Score	10							20							25					
Secondary Damage	**Laddering**					**Unravelling**											**Tearing combined with Laddering or Unravelling**			
Secondary Damage Penalty	10					20											30			
Penalty Score	=Secondary damage penalty*Hole enlargement resistance score/100																			

**Fig. 2:**
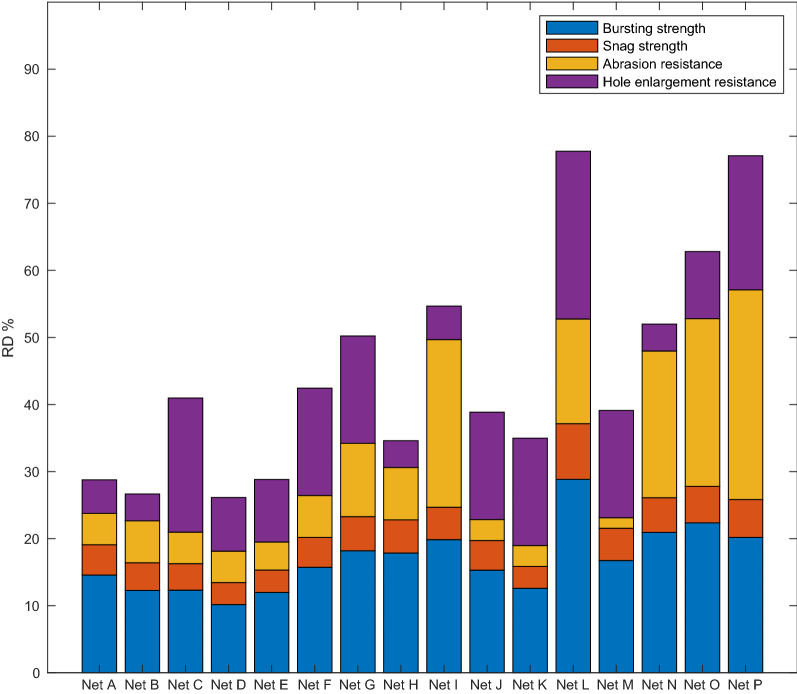
RD values for all LLINs following algorithm Method 1

The RD values for each LLIN calculated using Method 1 (Proximity to Aspirational Values) are reported in Fig. 2. Marked differences in RD values were observed across the different LLIN products, and none reached the aspirational value (RD = 100). Six LLINs achieved RD values of 50% RD (Net G, I, L, N, O and P), the remaining ten LLINs produced RD values of < 50.

**Fig. 3:**
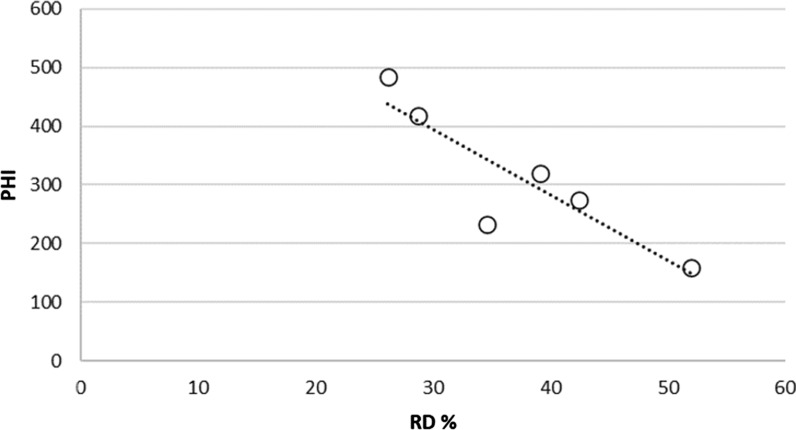
Method 1: PHI (field) vs. RD (lab) for six LLIN Products (r^2^ = 0. 78)

**Fig. 4:**
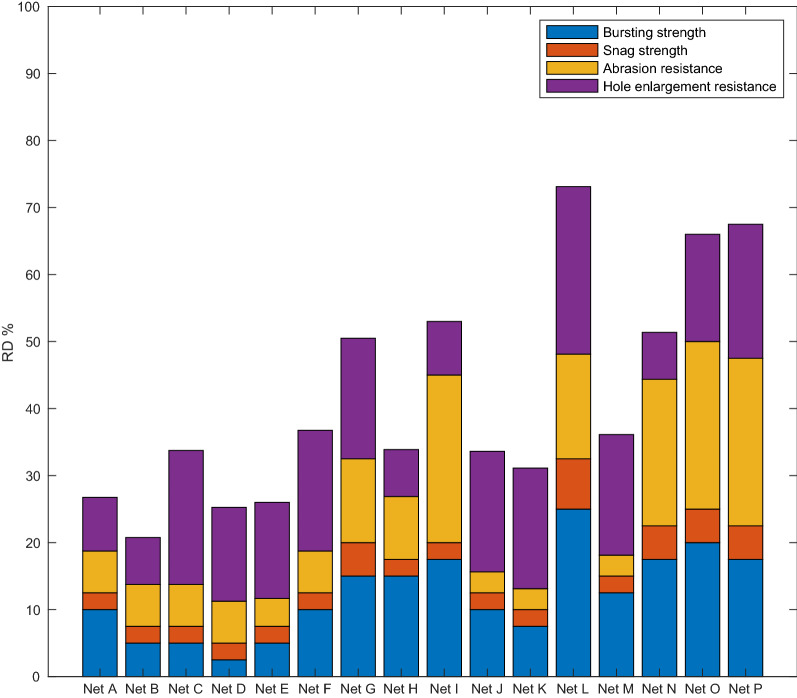
RD values for all LLINs following algorithm Method 2

These data also highlight marked differences in the performance of LLIN products in relation to each of the four damage mechanisms. The high resistance to abrasion of Net P and hole enlargement resistance in Net L are particularly noteworthy. The RD data (method 1) for six of the LLINs reported in Fig. 2 were compared with corresponding, separately reported PHI data for the same brands of net retrieved from the field after 1–3 years in use from the Wheldrake et al. study [13]. The results are shown in Fig. 3 and reveal an association between RD values obtained in the laboratory with PHI field data (r^2^ = 0.78, p < 0.05).

In relation to RD values calculated in accordance with Method 2 (Proximity to Aspirational Value by KPI), the same aspirational values were used as in Method 1 as defined in Table 7. The resulting RD values for each LLIN using Method 2 are given in Fig. 4. Generally, the resulting rank order of the RD values were very similar to those determined by Method 1 with six LLIN products achieving RD values ≥ 50% and none with RD < 20%.

**Fig. 5:**
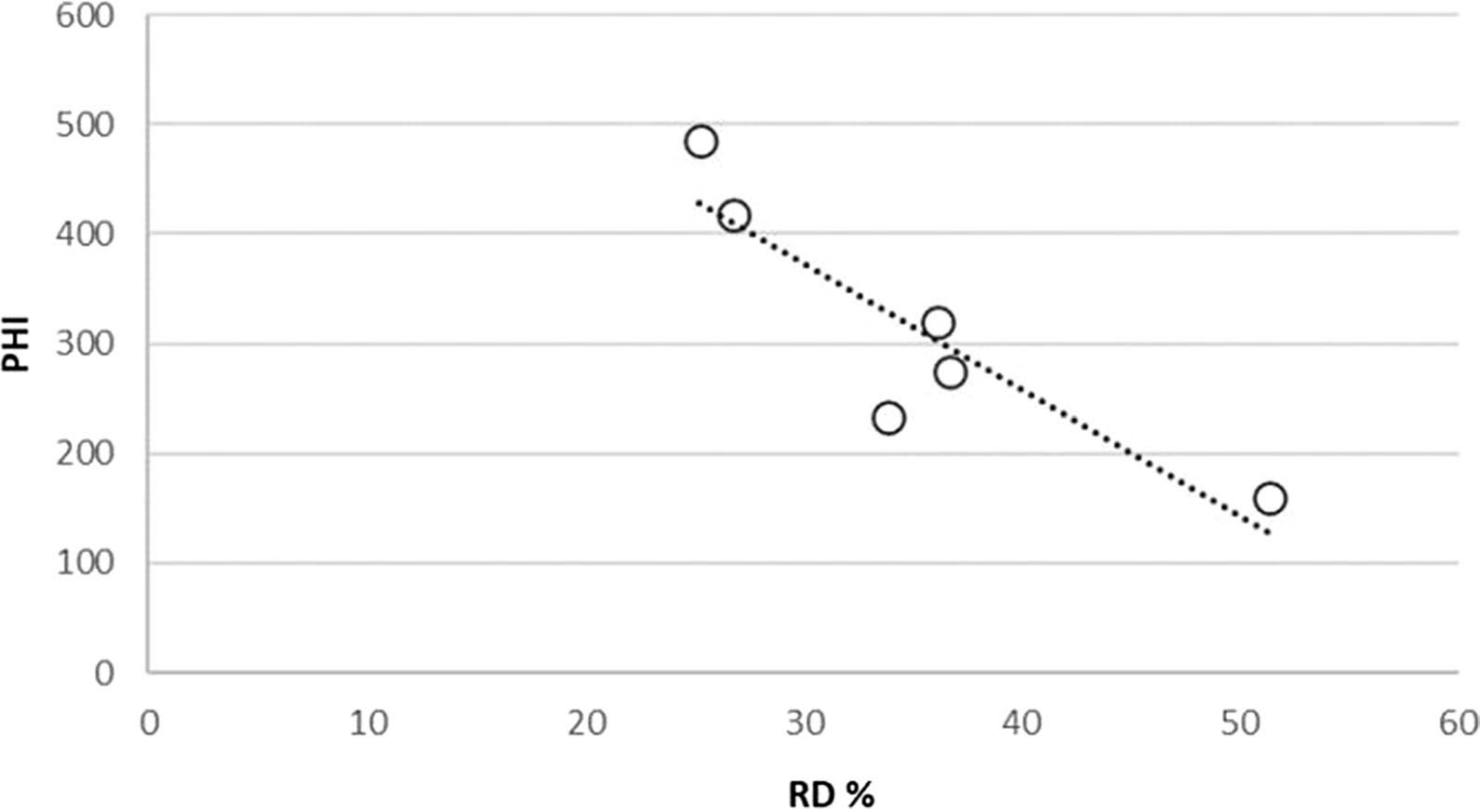
Method 2: PHI (field) vs. RD (lab) for six LLIN Products (r^2^ = 0.80)

**Fig. 6:**
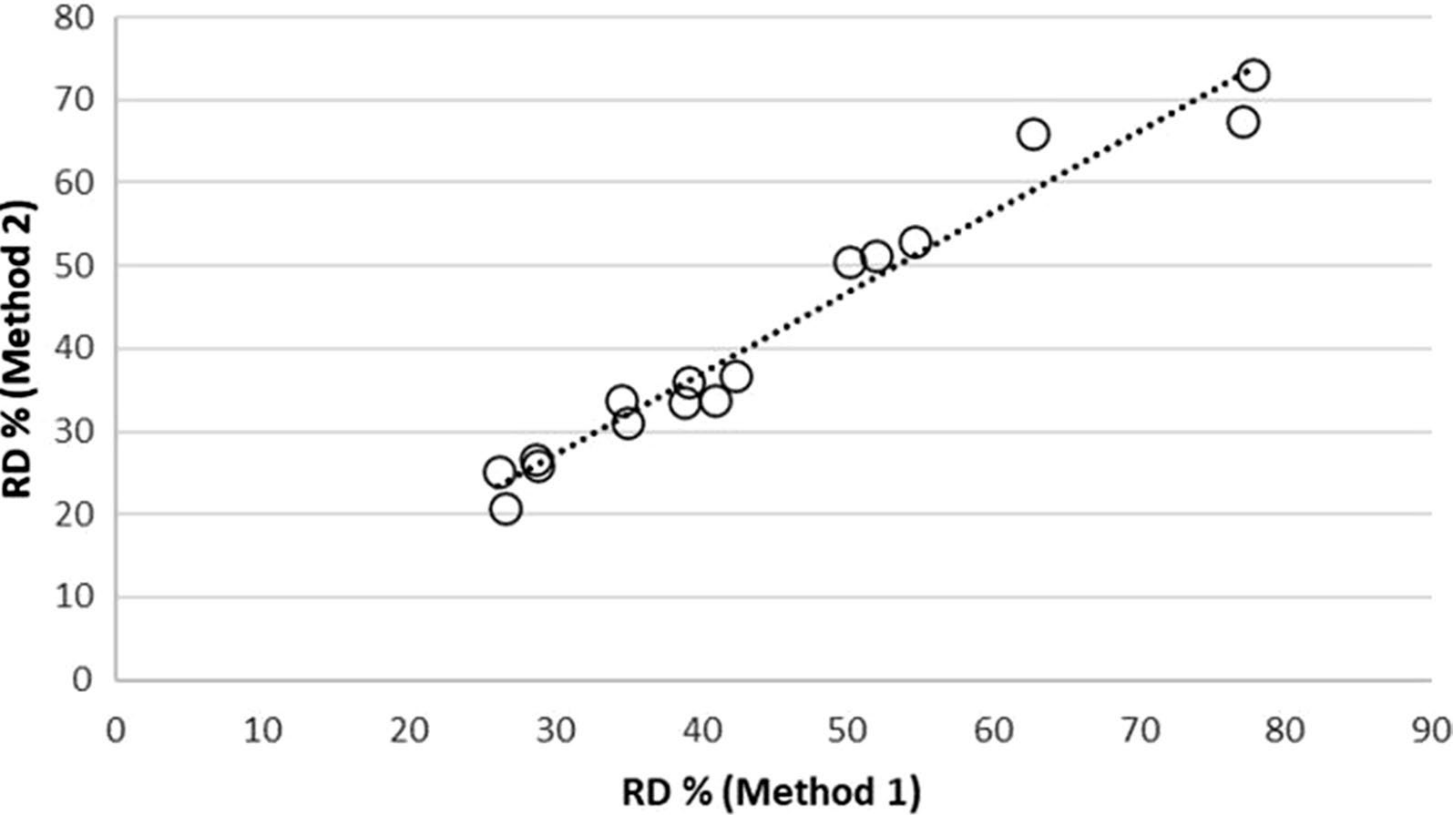
Correlation of RD values using Method 1 and Method 2 (r^2^ = 0.96)

As for Method 1, the results for six different branded LLINs retrieved from the field followed the same trend of reducing PHI with increasing RD using Method 2, see Fig. 5 (r^2^ = 0.80, p < 0.05).

Each method of calculating RD was capable of distinguishing LLINs that were more physically robust and capable of resisting damage than others and good correlation was observed between the outputs of both methods, Fig. 6 (r^2^ = 0.96, p < 0.05).

## Discussion

Resisting the major sources of damage LLINs are exposed to during normal use is essential if products are going to remain in good physical condition for many years. For years, the vector control community has relied upon measuring the bursting strength of LLINs in the laboratory to characterize the ‘physical strength’ of different products. However, these measurements are clearly insufficient given the root causes of hole formation [13]. In practice, there are multiple mechanisms of damage, but representing all in one meaningful resistance to damage (RD) metric would be more reliable. The approach described in the present work is distinct from other attempts to develop a single composite metric in that no field study evaluations are needed [30], and rapid assessment of any LLIN product is, therefore, possible prior to distribution. The RD approach focuses on measurements conducted under controlled laboratory conditions based on ISO procedures, and has the advantage that the inherent variations in field study evaluations are completely obviated.

In the RD methodology, the concept of aspirational targets has been introduced, taking into account the magnitude of forces LLINs are likely to encounter during normal use. Of course, the setting of these aspirational targets is open to contention, but at the very least, it focuses attention on what is actually needed in terms of functional performance. Based on an analysis of the real forces generated by human adults, a pragmatic approach was adopted for defining upper values for resistance to damage in terms of: bursting strength (700 kPa), snag strength (200 N), abrasion resistance (400 rubs) and hole enlargement (residual hole size < 5 mm corresponding to a score of 100 without laddering, unravelling or tearing). Note that the setting of aspirational targets needs to be considered not just in terms of product performance, but also in terms of economics. Generally speaking, engineering of LLINs capable of meeting high aspirational values are likely to improve cost effectiveness if LLINs are more robust for the entire life span.

The RD methodology also accounts for the fact that LLINs could be engineered to exceed aspirational targets in the future. Already, warp knitted nets are manufactured for use in other industries that would meet the targets suggested herein, albeit at significantly higher cost. For example, in sportswear applications, warp knitted fabrics with bursting strength values of > 2000 kPa are produced, albeit at heavier basis weights (> 90 g/m^2^) [31]. In Method 1, the actual proportion of the aspirational target is calculated, such that it is possible to score greater than 100 on the RD scale. This is clearly positive in terms of promoting product innovation. By contrast, in Method 2 the RD value is assigned based on a score matrix. The overall RD value is determined based on tiering of the individual parameter values in equal proportions. In this method it is possible for LLINs with snag strengths of e.g. 40 N to score the same as a net with snag strength of 59 N; similarly, this is possible with bursting strength and abrasion resistance. Therefore, this method does not enable scoring past RD = 100%. It is suggested that Method 1 is employed for future characterization of LLIN’s physical integrity. One could argue that Method 2 might encourage manufacturers to focus on meeting bare minimum testing requirements, rather than targeting higher performance.

The RD results calculated by both Method 1 and Method 2 demonstrate the scope for improvement in the design of LLIN products. In particular, focus is required to improve snag strength (red, Figs. 2 and 4), which is also the most common form of damage observed in the retrieved field nets [13]. Also, even in LLINs that are considered to be ‘strong’ in terms of their bursting strength, careful attention should be paid to resisting hole enlargement by unravelling or laddering of the fabric structure.

Assuming normal household use of a LLIN, that avoids interaction with rodents and exposure to naked flames, cigarettes, cooking embers or deliberate cutting with a knife, good correlation between PHI values in the field and calculated RD values from the lab has been observed, for both RD Methods 1 and 2. This supports the assertion that LLINs can be improved to better withstand certain types of common and reasonable mechanical damage, and this is unlikely to be resolved without upgrading product specifications. However, progressive improvements in RD scores, combined with behavioural change aimed at taking care of the product, is likely to have a major impact on long-term physical integrity and survivorship [32]. Although the physical integrity of the nets vary by location, a study carried out by Wheldrake et al. [13] showed that mechanical damage was the main contributor to hole formation across a range of geographies. Therefore, increases in the RD could improve the lifespan of LLINs across the board. Further field comparisons of RD scores and actual physical integrity are now needed to verify the suggested RD methodology as a valid approach to assess expected field performance of specific LLIN brands.

## Conclusions

The Resistance to Damage (RD) metric provides a reliable metric to characterize the physical robustness of LLINs prior to use based on simple laboratory testing of the textile material. Marked differences in RD scores between LLIN products were revealed, but none achieved the aspirational targets to maximize performance in use. Comparison of RD scores with PHI values from the field for the same LLIN brands suggests that higher RD (measured in the lab) is associated with reduced hole formation (in the field). There is significant scope for product innovation aimed at moving much closer to aspirational RD targets and improving relevant performance parameters.

## Abbreviations

WHO: World Health Organization
WHOPES: World Health Organization Pesticide Evaluation Scheme
PHI: Proportionate hole index
ITN: Insecticide-treated net
LLIN: Long-lasting insecticidal net
